# The Effects of Production System and Sex on the Sensory Quality Characteristics of Dorper Lamb

**DOI:** 10.3390/foods9060725

**Published:** 2020-06-02

**Authors:** Louwrens Christiaan Hoffman, Bianca Claasen, Daniël André Van der Merwe, Schalk Willem Petrus Cloete, Jasper Johannes Erasmus Cloete

**Affiliations:** 1 Department of Animal Sciences, Faculty of Agriculture and Forestry Sciences, University of Stellenbosch, Private Bag X1, Matieland 7602, South Africa; uaeclaasen@hotmail.com (B.C.); dvdmpok@gmail.com (D.A.V.d.M.); schalkc2@sun.ac.za (S.W.P.C.); jasperc@elsenburg.com (J.J.E.C.); 2 Centre for Nutrition and Food Sciences, Queensland Alliance for Agriculture and Food Innovation, University of Queensland, Coopers Plains, QLD 4108, Australia; 3 Western Cape Department of Agriculture: Directorate Animal Sciences: Elsenburg, Private Bag X1, Elsenburg 7607, South Africa; 4 Western Cape Department of Agriculture: Elsenburg Agricultural Training Institute, Private Bag X1, Elsenburg 7607, South Africa

**Keywords:** feedlot, free-range, sensory meat quality, lamb

## Abstract

The effect of production systems on the sensory quality characteristics of Dorper lambs was investigated. Sixty lambs (ewes, rams, castrates) were allocated into two production groups (feedlot or free-range) at weaning with equal numbers of each sex represented in each group. The lambs were fed for five (slaughter group 1) or six (slaughter group 2) weeks. Feedlot lambs were fed a commercial pelleted diet while free-range lambs utilized natural shrub pastures. Samples of the *Longissimus thoracis* muscle were used for sensory evaluation. Feedlot lambs produced meat that was juicier and more tender than meat from free-range lambs. Initial juiciness was also higher in the meat from the feedlot lambs. No aroma or flavour differences were observed. The meat from the free-range ram lambs (slaughter group 1) was the least tender, whereas the lamb flavour was also compromised in the free-range ram lambs. Free-range meat may not necessarily be distinguished from feedlot meat as far as aroma and flavour are concerned.

## 1. Introduction

Gastronomic satisfaction from the consumption of meat is mainly derived from tenderness, juiciness and flavour. Consumers consider meat tenderness the most important palatability trait [1], with some consumers expressing a willingness to pay a higher price for meat that could be certified as tender [2]. Juicy meat is also generally preferred [3]. Meat flavour contributes to overall product acceptability. These quality attributes need to be considered when raising animals to satisfy the consumer market.

A growing number of consumers have vocalised their preference for animal products of free-range origin [4]. Free-range products are perceived as natural, healthier and of higher quality than products from more intensive animal production systems [5,6]. Scientific investigations report differences between lamb carcasses from feedlot and free-range systems [7,8,9]. High planes of nutrition, often associated with feedlot diets, result in lambs that are generally fatter at slaughter compared to lambs on free-range diets. The fatter carcasses produced on feedlot diets show an improvement in meat tenderness through an increase in the intramuscular fat content relative to the muscle collagen [10]. Moreover, higher juiciness scores are associated with meat from lambs fed feedlot diets [11,12,13,14]. Fatter carcasses are also better insulated than lean carcasses, thus slowing down post-mortem chilling and improving meat tenderness by decreasing the extent of cold-induced muscle shortening. This also prolongs post-mortem proteolysis [15]. Animals that grow rapidly prior to slaughter, as is commonly found when high-quality feedlot diets are fed, produce tender meat [16]. The tenderness of meat is attributed to an increased protein turnover that results in higher concentrations of proteolytic enzymes in carcass tissues [17]. In addition, muscles from these faster growing animals tend to have less cross linked collagen [18]. In contrast, lambs fed low energy diets have also been reported to produce more tender meat than lambs fed a higher energy diet [19].

Some studies have shown that lamb sex has an effect on carcass composition, juiciness, tenderness and flavour [11,18]. In particular, intact male lambs produce leaner carcasses than castrates and ewes. Leaner carcasses are associated with less juicy and less tender meat compared to the fatter carcasses of ewes and castrate lambs [20,21]. Investigations on cattle did not detect significant differences in meat tenderness between bulls and steers. At the same age, meat from males is less tender than meat from females and this phenomenon is attributed to the higher calpastatin and μ-calpain activity at 24 h post mortem in meat from males [22]. According to Pommier et al. [23] testosterone levels in males increase after puberty, causing an increase in the quantity of collagen in the muscle, which reduces the tenderness of meat.

Flavour volatiles may be affected by the feeding system or the feed given [9,24,25]. Feedlot diets alter fat composition and reduce flavour intensity while free-range diets produce lamb with a more intense flavour [26]. However, Muir et al. [15] observed no flavour differences when free-range and feedlot lambs were slaughtered at the same weight and/or fat cover. In addition, high-quality pastures may also result in no flavour differences between free-range and feedlot meat [25]. Sheep meat odour and flavour are also affected by sex and its interactions with the feeding system [11,26,27].

Literature which compares the effect of the production/feeding system on the sensory meat characteristics of all three sex groups under southern African conditions is scarce. Most literature compares either the feeding system on the sensory meat quality characteristics between ewes and rams or rams and castrates. The Dorper sheep breed is the second most abundant sheep breed in South Africa and has been selected for its hardiness and adaptability under harsh conditions [28,29]. While lamb from Dorper sheep from different extensive grazing systems has been evaluated [24], the comparison of a free-range finishing regime with feedlot finishing for Dorper sheep under South African conditions has not yet been reported. In this study, Dorper lambs of all sex groups of the same age were finished off either in feedlot or in free-range conditions and slaughtered after a predetermined period. The main objectives of this study were to investigate the effect of the production system (free-range vs. feedlot) on the sensory characteristics of Dorper lamb, while the effect of sex on lamb sensory characteristics was studied as a secondary objective.

## 2. Materials and Methods

### 2.1. Experimental Outlay

Sixty Dorper lambs at the Nortier research farm (Coordinates: 32.035147078S, 18.331839395E) of the Western Cape Department of Agriculture were randomly sorted into two production groups, namely feedlot and free-range, subsequent to weaning. Each of the production groups consisted of 10 lambs from each sex class (ewes, rams and castrates). The lambs were allocated to these treatments at weaning (121 ± 5 days of age) at an average weight of 36.3 ± 3.2 kg. The feedlot lambs were fed a commercial pelleted diet while the free-range group grazed natural shrub pastures until slaughter. In the feedlot production groups, the lambs of different sexes were reared separately in three pens (10 m × 6 m), with 6 m^2^ space per lamb allowed. The different sexes of the free-range production group were in three adjacent and extensive grazing paddocks, with an area of 15 ha each, so as to ensure the lambs had access to similar grazing vegetation. Owing to logistic considerations, one group was slaughtered 5 weeks into the study while the others were slaughtered a week later. At each slaughter occasion, 15 lambs from both production groups were randomly selected (representing five lambs from each sex class). The experimental outlay (selection of the lambs, description of the production system, diet composition, plants grazed and selection of slaughter lambs) is detailed in the paper by Cloete et al. [30]. The samples from the lambs slaughtered at week 5 and week 6 were both included for the sensory analysis. The average live weights of the lambs at slaughter was 43.2 kg. This did not differ between the production groups. The slaughter weights of the rams (46.4 kg) and castrates (44.4 kg) on the other hand were heavier than those of the ewe lambs (38.7 kg).

Following slaughter, the carcasses were refrigerated for 48 h at 4 °C. The *Longissimus thoracis* (LT) muscles were then excised between the 8th to 11th ribs from both sides of each carcass. Excised LT muscles were placed in individually coded bags and transported in an insulated cool box to the Meat Science laboratory at Stellenbosch University (Stellenbosch, South Africa) for further analyses. The LT muscles from the right side of each carcass were used for subcutaneous fat thickness measurements [30]. The LT muscle samples from the left side of the carcass (with the subcutaneous fat layer still intact) were vacuum sealed and stored at −18 °C until required for sensory meat quality evaluation.

### 2.2. Preparation of Samples

Twenty-four hours before every sensory evaluation session, six LT muscles (one from each sex of each treatment) were removed from the freezer and defrosted in a refrigerator at 2−4 °C. After 24 h, each sample was removed from its packaging and placed on a metal rack (covered with foil paper with the reflective side up) in a coded oven bag (GLAD^®^). A thermocouple probe, connected to a handheld digital temperature monitor (Hanna Instruments, Johannesburg, South Africa) was inserted into the centre of each sample and the samples were roasted to an internal temperature of 68 °C in an oven preheated at 160 °C. The meat was roasted in two Defy ovens connected to a computerized electronic temperature system. At a core temperature of 68 °C, the meat samples were removed from the oven and allowed to cool for five minutes. All the visible fat and meat surfaces exposed to the cooking environment were trimmed. Each sample was cut into 1 cm^3^ cubes, perpendicular to the fibre direction. Thereafter, the cubes were individually wrapped in aluminium foil and reheated at 100 °C for 10 min before the commencement of each sensory evaluation session.

### 2.3. Sensory Analysis

The sensory panel consisted of seven trained assessors, between the ages of 40 and 60 years, previously selected for their flavour and texture sensitivity according to the guidelines of the American Meat Science Association (Nebraska, USA) [31]. The members of this sensory panel routinely volunteered to take part in sensory analyses; tipically more than half the panellists selected would have prior experience in analysing meat products. The panel was further trained using the consensus method described by Lawless and Heymann [32]. Attributes were rated on the basis of 100 mm unstructured lines with anchor points at each end, zero (left anchor point) representing the minimum and one hundred (right anchor point) representing the maximum. Scores were considered as the distance from the left anchor point. For all the sensory attributes analysed, except for flavour, a higher value depicted a better score. One sample from each sex group in each production system was used to train the panel for sensory attributes. The judges agreed on a consensus list of attributes for describing lamb which included the intensity of the lamb aroma, the impression of initial juiciness, the sustained juiciness, the impression of tenderness and the overall lamb flavour. Verbal definitions of the sensory attributes evaluated for the lamb are given in Table 1.

### 2.4. Sensory Procedure

The samples were served and evaluated during ten sessions. The panellists were seated in individual sensory booths in a controlled temperature room (natural day light at 22 °C). The meat samples (individually wrapped in aluminium foil) were placed in coded glass ramekins, preheated at 100 °C and presented in a completely randomized order as pertaining to production system and sex. The meat was evaluated using the standard questionnaire of the American Meat Science Association [31]. The aroma of the samples was assessed immediately after un-wrapping the aluminium foil. The initial juiciness was assessed by pressing the sample between the forefinger and the thumb. The sustained juiciness, tenderness and the flavour attributes were assessed by tasting the sample. For each session, the panel members were offered six different meat samples and the attributes were individually recorded. Wafer biscuits, apple slices and distilled water were used by the assessors to cleanse their palates between samples. The panellists were allowed 20 min for each testing session, with extra time being allowed if panellists needed more time to complete the testing.

### 2.5. Statistical Analysis of Data

The sensory data was subject to an analysis of variance (ANOVA) using the PROC GLM of the SAS version 9.1.3 statistical software [33] to evaluate the different sources of variation in the sensory attributes: lamb aroma, initial juiciness, sustained juiciness, tenderness and overall flavour. Production system, sex and slaughter date were the main effects and all the interactions between the main effects were considered. The Shapiro–Wilk test was performed to test for normality [34]. The sensory experiment consisted of three treatment combinations, each replicated ten times by seven panel members in a completely randomized design. The treatment combinations involved a 2 × 3 × 2 factorial design, arising from the combination of two production systems (feedlot and free-range) and three sex groups (ewes, castrates and ram lambs) and two slaughter dates (slaughter group 1 and 2). A *p*-value smaller than 5% (*p* < 0.05) was considered significant. Where applicable, Pearson’s correlations were calculated between the various sensory attributes and the physical and chemical characteristics reported in the paper by Cloete et al. [30].

## 3. Results

The analysis of variance (ANOVA) of the lamb attributes—lamb aroma, initial meat juiciness, sustained meat juiciness, tenderness and flavour—are presented in Table 2. None of the main effects of the production system, sex or slaughter group the affected lamb aroma. However, the two-way interaction between the production system and sex was significant for the lamb flavour. Slaughter date had no effect on any of the sensory attributes except tenderness where a three-way interaction between the production system, sex and the slaughter date, was significant.

Meat from the lambs slaughtered on the two slaughter dates did not differ with respect to any of the sensory traits evaluated in Table 3 (*p* > 0.05). Meat from the feedlot lambs tended (*p* = 0.064) to have a higher initial juiciness (Table 3). Significantly higher sustained juiciness scores were also observed for meat from feedlot lambs compared to the meat from the free-range lambs (*p* = 0.015). A positive correlation was observed between the initial juiciness and the sustained juiciness (*p* < 0.05; *r* = 0.800) while a negative correlation was present between the initial juiciness and the drip loss (*p* < 0.05; *r* = −0.54) as well as between the initial juiciness and the cooking loss (*p* < 0.05; *r* = −0.418). The traits reported in Table 3 were unaffected by sex (*p* > 0.05).

The three-way interaction between the production system, sex and the slaughter date, was significant (*p* < 0.05) for tenderness (Table 4). The meat from ewe lambs tended to be more tender than the meat from castrates although the difference was not significant. The meat from free-range ram lambs slaughtered on slaughter date 1 (denoted as FR-ram-1) had the lowest tenderness scores.

A two-way interaction (*p* < 0.05) between the feeding system and the sex (Table 5) was observed for flavour. Free-range ram lambs scored the lowest for flavour while the free-range ewe lambs scored the highest. It was, however, noted that all the groups scored above 80 on a 100 mm scale.

## 4. Discussion

It is well known that initial meat juiciness is related to the water holding capacity (WHC) of meat. Higher WHC values indicate higher initial juiciness scores whereas lower WHC values indicate lower initial juiciness scores. This is reflected by the negative correlation found in this study between the initial meat juiciness and drip loss, (*p* < 0.002; *r* = −0.54), where higher levels of drip loss are indicative of a low WHC of meat. Sustained meat juiciness depends on the amount of liquid released during mastication, both from the food and saliva. The higher intramuscular lipid content of the meat from feedlot lambs (in absolute terms) compared to that of free-range lambs (3.64% vs. 3.43%, respectively) [30] might have affected the perception of meat juiciness (*p* < 0.05; *r* = 0.43) by stimulating saliva flow.

The less tender meat of free-range lambs may be attributed to the higher level of exercise of these lambs during grazing activity [35]. This result is in agreement with that of Vestergaard et al. [36] who also suggested physical activity as the reason for the lower tenderness scores observed in bulls raised on pastures compared to the tie-stalled bulls that were fed a concentrate-based diet. Lowe et al. [37] did not find any significant differences in tenderness between free-range and feedlot lambs, based on mechanical shear force values, possibly because nutrition was adequate and stress levels were low in their experiment.

It is known that at the same age, meat from male lambs is less tender than that from female lambs (Table 4) [22,38]. This is the result of an increase in the excretion of testosterone in males after puberty, leading to an increase in the amount of collagen in muscles, which in turn, results in reduced tenderness [23,39]. Other research results indicate that meat from male animals have a higher calpastatin activity 24 hr post-mortem which may decrease the quantity of myofibrillar protein proteolysis thus resulting in less tender meat [22]. Furthermore, Pommier et al. [23] also attributed less tender meat from males at the same age, compared to ewes or castrates, to testicular hormones. Studies involving beef cattle also showed that meat from bull carcasses was less tender and hence less palatable than meat from steer carcasses [20,21,40,41]. However, some investigations have been unable to detect significant differences between the meat from bulls and steers [42]. As mentioned earlier, intact males generally produce leaner carcasses (than ewes and castrates) that are associated with less tender meat [20,21,40]. In addition, low energy diets such as free-range diets are often associated with less tender meat although contradictory results have been reported [19,43]. Rousset-Akrim et al. [26] accordingly showed that lambs raised under free-range conditions had a more intense flavour than lambs raised under feedlot conditions although their difference applied across sex groups.

## 5. Conclusions

The results from this investigation indicated that the production system affects some sensory characteristics of Dorper lambs. The advantage of feedlot-feeding appears to be its ability to produce juicier meat compared to free-range-feeding—a phenomenon attributed to the higher lipid content of the former. Meat tenderness was compromised in ram lambs in the free-range production system for lambs slaughtered in slaughter group 1 when the production system by sex by slaughter date interaction was considered, an observation which is difficult to explain. Overall, the production system had no effect on the aroma of Dorper lamb. However, lamb flavour was more intense in ram lambs from the free-range production system when the feeding system by sex interaction was considered. Meat tenderness was evidently compromised in ram lambs compared to the other sex groups. As the organoleptic differences between the two production systems were minimal, other social cues such as carbon footprint, perceived animal welfare, etc. may play a role in influencing the consumer selection of the production system’s lamb that they would consume—this warrants further research.

## Figures and Tables

**Table 1:** Verbal definitions used to describe the sensory attributes of the Dorper lamb.

Attribute	Definition	Scale
Lamb aroma	Aroma associated with lamb	0 = No lamb aroma 100 = strong lamb aroma
Initial juiciness	The amount of fluid that exudates on the cut surface when pressed between thumb and fore finger	0 = extremely dry 100 = extremely juicy
Sustained juiciness	The degree of juiciness perceived after the first two to three chews between the molar teeth	0 = extremely dry 100 = extremely juicy
Tenderness	Impression of tenderness after the first two to three chews between the molar teeth	0 = extremely tender 100 = extremely tough
Flavour	Flavour associated with lamb as a combination of taste and swallowing	0 = bland flavour 100 = intense flavour

**Table 2:** Analysis of variance (ANOVA) for the attributes: aroma; initial juiciness; sustained juiciness; tenderness; and flavour for Dorper lambs (rams, ewes, castrates) reared either under free-range conditions or in a feedlot system.

Source	dF	Lamb aroma		Initial Juiciness		Sustained Juiciness		Tenderness		Flavour	
		MS	*p*-value	MS	*p*-value	MS	*p*-value	MS	*p*-value	MS	*p*-value
Production system	1	32.89	0.350	289.63	0.064	486.27	0.015	549.38	0.024	2.67	0.599
Sex	2	48.35	0.280	22.40	0.756	122.15	0.211	640.02	0.004	35.77	0.031
Slaughter date	1	74.41	0.160	96.03	0.278	169.69	0.141	145.80	0.234	0.02	0.965
Production system × Sex	2	16.99	0.640	175.81	0.123	18.95	0.779	137.59	0.264	46.09	0.013
Production system × Slaughter date	1	0.01	0.990	0.04	0.982	0.49	0.936	3.67	0.850	0.74	0.780
Sex × slaughter date	2	36.50	0.380	30.96	0.680	7.64	0.900	10.53	0.900	7.77	0.450
Production system × Sex × Slaughter date	2	35.50	0.390	53.81	0.514	7.73	0.903	347.62	0.040	15.37	0.210

MS denotes the mean squares of the attributes; dF = degrees of freedom.

**Table 3:** Means (±s.d.) depicting the effect of the slaughter date, production system and the sex on the sensory attributes of the Dorper lamb.

Attribute	Slaughter Date		Production System		Sex		
	1	2	Feedlot	Free Range	Castrates	Ewes	Rams
Aroma	81.83 ^a^ ± 8.91	83.14 ^a^ ± 7.92	82.82 ^a^ ± 8.52	82.15 ^a^ ± 8.37	82.17 ^a^ ± 8.07	83.02 ^a^ ± 8.76	82.26 ^a^ ±8.52
Initial juiciness	81.72 ^a^ ± 8.16	84.25 ^a^ ± 7.08	83.94 ^y^ ± 7.58	82.02 ^z^ ± 7.80	83.25 ^a^ ± 7.42	83.70 ^a^ ± 7.95	82.33 ^a^ ± 7.86
Sustained juiciness	79.38 ^a^ ± 9.31	81.63 ^a^ ± 8.69	81.67 ^a^ ± 8.82	79.33 ^b^ ± 9.18	80.75 ^a^ ± 8.36	81.38 ^a^ ± 9.88	79.40 ^a^ ± 8.84

^a,b^ Different subscripts within a main effect differ at *p* < 0.05. ^y,z^ Different subscripts within a main effect tend to differ at *p* < 0.10.

**Table 4:** Means (±s.d.) depicting the effect of the three-way interactions (production system × sex × slaughter date) on the tenderness scores of Dorper lambs.

Production System × Sex × Slaughter Date	Tenderness
FL-castrate-1	87.87 ± 8.02
FL-castrate-2	85.57 ± 9.00
FL-ewe-1	89.04 ± 6.62
FL-ewe-2	87.67 ± 9.47
FL-ram-1	84.83 ± 8.90
FL-ram-2	85.33 ± 6.31
FR-castrate-1	85.31 ± 8.66
FR-castrate-2	87.48 ± 8.08
FR-ewe-1	86.50 ± 7.75
FR-ewe-2	84.87 ± 10.03
FR-ram-1	79.38 ± 8.24
FR-ram-2	81.73 ± 8.43

Where FL and FR refer to the production systems, feedlot and free range, respectively. The sexes are denoted by castrate, ewe and ram, while the slaughter date is denoted as either 1 or 2.

**Table 5:** Means (±s.d.) depicting the effect of the two-way interaction (production system × sex) on the flavour score of Dorper lambs.

Production System × Sex	Flavour
FL-castrate	82.37 ^a^ ± 7.92
FL-ewe	82.64 ^a^ ± 8.77
FL-ram	82.71 ^a^ ± 8.81
FR-castrate	82.95 ^a^ ± 7.56
FR-ewe	82.98 ^a^ ± 8.32
FR-ram	80.98 ^b^ ± 7.39

^a,b^ Different subscripts differ at *p* < 0.05. FL and FR refer to the production systems, feedlot or free range.
